# Menstrual Disorders Following Covid 19 Vaccination in Women of Reproductive Age and Post‐Menopause. A Systematic Review

**DOI:** 10.1002/hsr2.71103

**Published:** 2025-08-19

**Authors:** Farideh Pargar, Zeinab Rabiei, Afsaneh Keramat

**Affiliations:** ^1^ Student Research Committee, School of Nursing and Midwifery Shahroud University of Medical Sciences Shahroud Iran; ^2^ Department of Midwifery, School of Nursing and Midwifery Bushehr University of Medical Sciences Bushehr Iran; ^3^ Center for Health Related Social and Behavioral Sciences Research Shahroud University of Medical Sciences Shahroud Iran

**Keywords:** covid ‐19, menstrual disorder, post‐menopause, reproductive age, vaccination, women

## Abstract

**Background:**

During the coronavirus pandemic, several factors such as stress, depression, infection, and vaccination against the COVID‐19 virus have caused changes in the menstrual cycle.

**Objective & Aims:**

This systematic review intended to provide a comprehensive interpretation of the changes in the menstrual cycle of women of reproductive age and menopause after coronavirus vaccination. Method: Electronic databases, including Web of Science, PubMed, Scopus, ScienceDirect, ProQuest, Cochrane, SAGE, Springer, Google Scholar, and CINHAL were searched for published studies from March 2020 to June 2023. Of the 682 references identified in the initial search, 27 studies met the inclusion criteria.

**Results:**

The results of this systematic review showed that COVID‐19 vaccination is associated with a wide range of menstrual disorders in women of reproductive age and postmenopausal women. The most commonly reported disorders were menorrhagia, delayed menstruation, changes in menstrual cycle length, spotting between periods, and increased bleeding volume. In postmenopausal women, complications such as spotting and resumption of bleeding were also observed. Although the prevalence and incidence of each disorder varied across studies, these findings suggest that vaccination can lead to changes in the menstrual cycle.

**Conclusion:**

COVID‐19 vaccination may lead to menstrual disorders in women of reproductive and postmenopausal age, which can cause concern and reduce social and psychological quality of life. However, these effects are usually temporary and transient, resolving after a few menstrual cycles. Further studies are needed to investigate the possible mechanisms of this association and identify associated risk factors.

## 1 Introduction

Following the outbreak of the Covid‐19 pandemic, various vaccines to prevent this dangerous disease were approved in December 2020 [1]. Researchers have shown that the COVID‐19 vaccination can protect against the deadly and severe consequences of the disease. However, some people are not sure about the preventive effect of this vaccine [2]. In 1913, a doctor in New York published a study and claimed that vaccination affects the quality, quantity, and regularity of menstrual bleeding, and showed a significant relationship between typhoid vaccination and menstrual changes among hundred cases [3]. Also, HPV vaccination was reported to cause menstrual disorders [4]. Concerns and unfounded information concerning COVID‐19 vaccination impairing reproductive health have also become a significant reason for vaccine mistrust in the general population and vaccine hesitancy among children, adolescents, menopause, and women of reproductive age [5]. Various types of vaccines approved by the FDA Organization are approved and used all over the world. These vaccines are divided into three main groups. The first group of HAS vaccines is based on mRNA, which includes two categories such as (Pfizer and Moderna), the second group is inactive viral vaccines, which include (Sinopharm and Sinovac) and the third group is based on the newly synthesized adenoviral vector (Johnson& Johnson/Janssen, Oxford‐AstraZeneca, and Sputnik). Vaccines function by mobilizing the immune system to protect from disease if exposure occurs [6, 7]. This immune activation is important, although it may also produce other localized or systemic [8, 9]. The result of research conducted in 2020 showed that the inner lining of the uterus (endometrium) is resistant to infection caused by the coronavirus because this lining has lower levels of ACE2, the receptor that the coronavirus binds to [9, 10] Although other studies have shown that the stress caused by any infectious and noninfectious disease can affect the normal menstrual cycle. It can disrupt the hypothalamic‐pituitary‐ovarian (HPO) axis that regulates menstruation. Increased cortisol levels can affect the production and balance of sex hormones. Change in the level of hormones lead to menstrual irregularity [11, 12, 13]. According to current evidence and reports, the hypothesis that menstrual disorders are caused by stress or are side effects of COVID‐19 vaccination cannot be rejected with certainty [14, 15]. The decrease in the number of infected people and the number of deaths should not have convinced people that the coronavirus is over. There are several uncertainties about the end of Corona. One of them is the uncertainty in the duration of the immunity of the infected, and the other is the creation of new strains that may require more booster doses [16]. The purpose of this systematic review study was to comprehensively review the disturbances of menstrual cycle changes after the COVID‐19 vaccination and provide significant scientific achievements for doctors and consultants. Also, it can answer the stress and worries of menopausal and reproductive‐age women and their fear of infertility in the future.

## 2 Methodology

### 2.1 Study Design and Population

**Figure 1:**
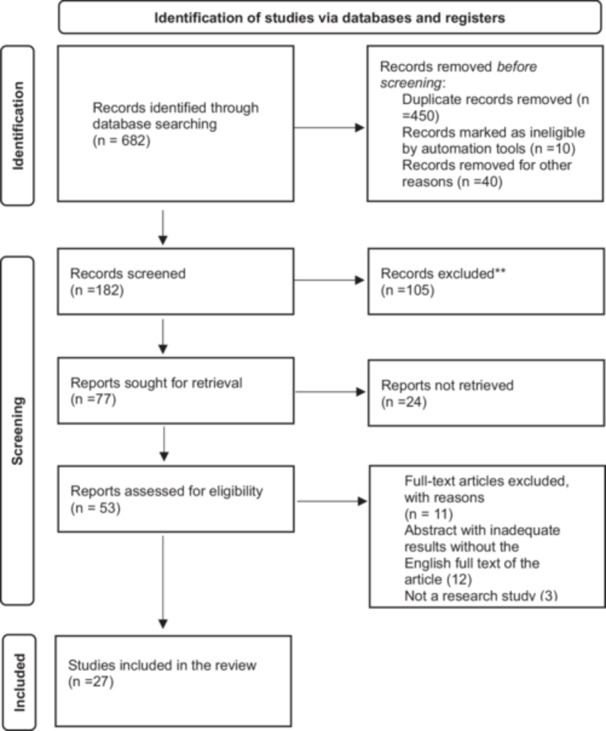
PRISMA flowchart.

Two independent authors (A.K. and Z.R.) searched for published studies from March 2020 to January 2021 using the following keywords. Two subsets of keywords were generated: [1] Covid‐19 and vaccine types with their manufacturing technologies and mode of action (e.g., COVID‐19 and mRNA vaccines (Pfizer and Moderna), vector‐borne vaccines (e.g., AstraZeneca and Johnson & Johnson), protein subunit vaccines (Novax), inactivated viral vaccines (Sinopharm), and adenovirus vaccines (Sputnik) and their side effect [2] vaccine types and adverse menstrual disorders (e.g., “women health” “women‐related problems” “menstrual irregularities” “menstrual problems” menstrual change, menstrual irregularities, menstrual disturbance, menstrual condition menstrual period, menstrual cycle characteristics “dysmenorrhea “menorrhea, “metrorrhagia” “amenorrhea” “oligomenorrhea” “hypomenorrhea” premenstrual syndrome and resumption ministration in menopause women). These subsets were systematically combined using Boolean operators (AND, OR, NOT) across electronic databases, including Scopus, Web of Science, PubMed, ProQuest, ScienceDirect, Cochrane, SAGE, Springer, and Google Scholar. It was not used due to Mesh term mismatches in all databases. The results were downloaded from all given combinations into the Covidence library for review. The search strategy is shown in Figure 1.

### 2.2 Inclusion and Exclusion Criteria

Studies were eligible for inclusion if they were original articles, published full‑text articles in English languages, and evaluated menstrual disorders after vaccination in reproductive and postmenopausal women. Studies were excluded if they were a review of the literature, were abstracts with incomplete results, and the full text was not available, or non‐English language publications were excluded.

### 2.3 Data Extraction and Assessing Evidence Certainty

The datum extracted by two independent authors (F.P. and Z.R.) extracted several features from the included studies, such as the author's name, year of publication, objectives, study population, sample size, study design, sampling size, and outcome. Any discord between the three reviewers was resolved through discussion with the senior author (A.K.).

The method of measuring the quality of observational studies was done using a standard Ottawa—new measure and the articles with the quorum score were included in the study.

### 2.4 Findings

Figure 1 PRISMA diagram shows that 682 studies were identified through comprehensive electronic searches via initial searching. Ultimately, we considered 27 observational articles (9 cohorts 11 cross‐sectional, and 7 retro perspectives) that qualified for the analysis with 196,011 participants based on the inclusion criteria. Menstrual disorders such as menorrhagia, oligomenorrhoea, metrorrhagia, and dysmenorrhea, hypomenorrhea polymenorrhea, and delayed menstruation were observed. The most common disorder reported was menorrhagia and delay in menstruation. Postmenstrual disorders, spotting, and resumption of menstruation were also seen in postmenopausal women, and no serious disorders were reported, the summary of the study characteristics is shown in Table 1.

**Table 1:** Main characteristics of the studies included in the present systematic review.

Authors (Year)	Study population	Sample size	Study design	Kind of vaccines	Menstrual changes related to the type of vaccine	Conclusion
Luisa [21] (2021)	18–41	*N *= 184	Cross‐sectional	1‐Moderna 2‐Pfizer 3‐ Janson 4‐Sinovac 5‐AstraZeneca	Fequency (normal 43.47%, low 25%, frequent 31.53%), regularity of cycles (irregular 42.93%, regular 8/51%) duration (normal 65.21%, long term 26.08%, amenorrhea 8.69%) amount (heavy 41.84%, light 20.65%, and amenorrhea 5.65%).	More studies and patient background information are needed to prove the complications caused by vaccination. This information should include patterns related to menstrual health (menstrual irregularity, dysmenorrhea, constipation, premenstrual syndrome, and mental, psychological, and spiritual conditions.).
Bing Zhang [32] (2022)	18–45	*N* = 13118	Retrospective study	1‐Moderna 2‐Pfizer 3‐ BioNTech 4‐Janson	In the Covid‐19 vaccine group (35.26%) and (28.33%) in the non‐Covid‐19 vaccine group delayed menstruation (20.57%), intermenstrual bleeding (15.92%) menorrhagia (0.21%).	Vaccination may lead to some disorders and menstrual irregularities in women. Findings and results are likely to be affected by the accuracy, quality, and number of reports, as a result, investigating side effects and menstrual disorders that are directly and transparently related to the COVID‐19 vaccine is somewhat challenging and controversial. A significant relationship was observed in this study.
Katharine Lee [33] (2022)	18–45 year 54–46 year And > 55 years	*N *= 39129	Retrospective study	1‐Moderna 2‐Pfizer 3‐ Janson	Menorrhagia (42%) and 51% of postmenopausal people also had metrorrhagia.	The most disorder was in the age group of 25–34 years and related to the Pfizer vaccine.
Laura Baena [22] (2022)	18–55	*N* = 14153	Cross‐sectional	1‐Moderna 2‐Pfizer 3‐ Janson 4‐AstraZeneca	Menorrhagia (43%) Dysmenorrhea (41%) Delayed menstruation(38%) Polymenorrhagia (32%)	Women vaccinated against COVID‐19 commonly experience menstrual and premenstrual changes.
Alexandra Alvergne [3] (2022)	18–40	*N* = 79	Cohort study	1‐Moderna 2‐Pfizer 3‐ Janson 4‐AstraZeneca	Menorrhagia (11.5%) Metrorrhagia (6.1%) Polymenorrhagia (1.5%).	Menstrual disorders and more irregularities may appear later and over time, and the findings of this study do not have enough power in the time frame of examining disorders to claim this possibility. There is no statistically significant relationship.
Siwan Wang [22] (2022)	> 18	*N* = 3527	Retrospective study	1‐Moderna 2‐Pfizer 3‐ Janson	Menstrual cycle disorders are showed.	Vaccinated women had a higher risk of increased cycle length than unvaccinated women (odds ratio, 1.48; 95% confidence interval, 1.00–2.19). Covid‐19 vaccination can cause changes in the menstrual cycle, especially in people who have previously had menstrual disorders. And among women whose cycles were short, long, or irregular before vaccination, the odds ratio is 1/10 times higher than women who had regular periods.
Alison Edelman [34] (2022)	18–45	*N *= 19622	Retrospective cohort study	1‐Moderna 2‐Pfizer 3‐Janson	There were no significant disorders, although the statistical relationship was significant.	Changes in cycle length were less than 1 day after the first and second vaccination. Changes during the menstrual cycle are not related to the type of vaccine.
Victoria Male [4] (2021)	> 18	*N *= 2241	Retrospective cohort study	1‐Moderna 2‐Pfizer 3‐ Janson 4‐AstraZeneca	There is no statistically significant relationship.	A particular type of vaccine is not associated with menstrual changes or menstrual flow. Vaccination in the last 2 days of the menstrual cycle, and having a history of uterine fibroids and menstrual disorders did not lead to a change in the flow of bleeding or a change in the timing of menstruation.
Antonio Simone[17] (2022)	15–45	*N *= 164	Cross‐sectional	1‐Pfizer 2‐Janson 3‐AstraZeneca	Menorrhagia (55.6%) Hypomenorrhea (11.1%) Polymenorrhagia (11.1%).	Most of the disorders and side effects occurred in women who received the AstraZeneca vaccine. Regardless of the type of vaccine, menstrual cycle disturbances occur in 50%–60% of cases after the first dose and in 60%–70% of cases after the second dose. Most of these women reported that they had their period 1 to 5 days earlier than expected and the calendar date of their menstrual cycle, and this disorder mostly occurred when the first dose of vaccine was injected in the first 14 days of the menstrual cycle or follicular phase.
Muhammad Sualeh [33] (2022)	18 >	*N* = 245	Cross‐sectional	All types of vaccines	Polymenorrhagia (21/4%) Menorrhagia (15/1%) Gotten worse in Menstrual symptoms (35/3%).	Vaccination is likely to change the length of menstruation, amount of bleeding, regularity, frequency and any associated symptoms. These changes may affect women's daily activities and reduce their overall quality of life. On the contrary, women whose menstrual pattern was normal after vaccination had less menstrual disorders after vaccination.
Işılay Taşkaldıran [35] (2022)	18–50	*N *= 573	Cross‐sectional	1‐Pfizer 2‐AstraZeneca 3‐sinovac	Polymenorrhagia (3/7%) Oligomenorrhea (5/7%) Metrorail (2/4%) Menorrhea (3/7%).	15.1% claimed that there were changes in their menstrual pattern in the first 3 cycles after vaccination. Changes in menstrual pattern after the first dose of vaccination 43.3% after the second dose, 33.3% after the third dose, 21.7% after the fourth dose, 1.7%.
Luisa Rodríguez [35] (2022)	18–44	*N *= 184	Retrospective study	1‐Moderna 2‐Pfizer 3‐Janson 4‐AstraZeneca 5‐sinovac 6‐Other	Frequency [regular (43/47%) polymenorrhagia (31/53%) oligomenorrhea (25%)] regularity of cycles [regular (51/8%), irregular (42/93%), amenorrhea (5/97%)] volume [heavy (41/84%), light (20/65%, amenorrhea (6/52%)].	SARS‐CoV‐2 infection and COVID‐19 vaccination can affect the menstrual cycle and cause changes.
Karen K Wong [36] (2022)	> 18	*N *= 62679	Cohort study	BNT162b2 mRNA‐1273 Ad26.COV2. S Other/unknown	Timing of menstruation (83/6%) severity of menstrual symptoms (67%) post‐menopausal bleeding (4%) Resumption of menstruation (2/8%).	51.9% of those who reported menstrual irregularity or vaginal bleeding, had received MRNA vaccine or BNT162b. 48.2% of these women were in the age range of 49–85 years.
Azza Sarfraz [37] (2022)	18–45	*N* = 510	Cross‐sectional	All types of vaccines	About 66.3% of participants reported menstrual symptoms postvaccination, of which 46.7% experienced them after their first dose. However, in 93.6% of participants, the symptoms resolved within 2 months. Vaccine type did not significantly influence the incidence of abnormalities (*p* > 0.05).	Vaccinated women had a higher risk of change during the period between periods than unvaccinated women. It is necessary to explain to women receiving the covid‐19 vaccine that menstrual irregularities may be expected. For women who are trying to get pregnant or are postmenopausal, it is important to be aware of these menstrual irregularities.
Elizabeth A [38] (2022)	20–> 50	*N *= 9652	Cohort Study	1‐Moderna 2‐Pfizer 3‐Janson	Lengthening of the cycle following the mRNA vaccine (0/97 day). After second‐dose cycles (1.43 days) Lengthening of the cycle following the Sigle dose J&J 1/26 days.	Compared to pre‐vaccination cycles, follicular phase vaccination with mRNA vaccine was associated with increased mean cycle length in first‐dose cycles. Vaccination with mRNA vaccine during the follicular phase compared to cycles before vaccination was associated with longer and longer menstrual cycles in the first cycles after injection.
Trogstad L [39] (2022)	18–30	*N *= 5688	Cohort study	1‐Pfizer 2‐BioNTech 3‐ChAdOx1	Dysmenorrhea (14.6%) Menorrhagia (13.6%) Polymenorrhagia (12%).	The menstrual disorder was observed regardless of the type of vaccine. There is a significant increase in menstrual changes after vaccination, especially for heavier bleeding than usual, longer duration and for shorter intervals between periods. Pregnancy disorders increase significantly after vaccination. including heavier bleeding, longer periods and shorter intervals between periods. The risk of severe bleeding after the second dose of the vaccine was 65.7%.
Mosini G [40] (2022)	18–45	*N* = 261	Cross‐sectional study	1‐Pfizer 2‐Moderna	Amenorrhea (7%) menstrual cycle abnormal‐menorrhagia (6%) Metrorrhagia (5%) statistical relationship was not significant.	87.7% of menstrual disorders were due to Pfizer vaccination and 14% were due to Moderna. More studies are needed to prove the relationship between covid‐19 vaccination and menstrual disorders vaccine. 78.6% of the cases did not report serious disorders.
Alvergne A [23] (2022)	18–40	*N *= 1729	Cross‐sectional	1‐Moderna 2‐Pfizer 3‐Janson 4‐sputnik	More disorders were in the younger age (15–25 year) group and were related to the mRNA vaccine. Delayed onset of menstrual bleeding (32.54%, first dose; 30%, second dose.	40% of women reported menstrual disorders. (After receiving the first dose (45%) or second vaccine dose (36%).
Alexandra alvenger [3] (2021)	18–45	*N *= 4989	Retrospective study	1‐AstraZeneca 2‐Pfizer 3‐BioNTech	Menorrhagia (11.5%) Metrorrhagia (6.1%) Polymenorrhagia (1.5%).	Menstrual changes in 20% of women may occur for a longer period of time after vaccination. This study does not have the necessary and sufficient time depth to prove this claim.
EeVon Woon [41] (2022)	> 18	*N *= 79	Cohort study	1‐Moderna 2‐Pfizer 3‐AstraZeneca	Timing of bleeding— menstrual flow— premature menstruation (0/47 day) deluded menstruation (0/3 day).	No significant changes were seen after vaccination.
Najla Dar [42] (2022)	22–71	*N* = 348	Cohort study	1‐Pfizer 2‐AstraZeneca 3‐Pfizer	Nearly 8% of females in the reproductive age reported menstrual abnormalities and tiredness.	The results of this study have reported menstrual disorders after vaccination of Adenovirus and mRNA. Although the menstrual changes were transient and short‐term, there were no reports of infertility disorders.
Nadia Muhaidat [42] (2022)	14–54	*N *= 2269	Cross‐sectional	1‐Pfizer 2‐AstraZeneca 3‐sinovav	Menorrhagia (19.46%) Polymenorrhagia (18.18%) Metrorrhagia (2.2%) Dysmenorrhea (21.25%) Worsening premenstrual symptoms (3.92%).	About 66.3% of females reported menstrual changes postvaccination, of which 46.7% experienced them after their first dose. However, in 93.6% of participants, the symptoms resolved within 2 months. kind of Vaccine did not significantly influence the incidence of abnormalities (*p* > 0.05).
Abdul Azeez [43] (2022)	18–> 65	*N *= 969	Cross‐sectional	1‐Moderna 2‐Pfizer 3‐ Janson 4‐sputnik 5‐sinovac	Menstrual disorder at 0.5%.	One of the complications identified in this study is menstrual disorders.
Ma'mon M (2022) [41]	20–39	*N *= 10064	Cohort study	1‐Moderna 2‐Pfizer 3‐ Janson 4‐sputnik 5‐sinovac 6‐AstraZeneca	Menstrual dysfunctions About 72% of participants experienced side effects after vaccination.	Nearly 88% of the participants were vaccinated with one of three COVID‐19 vaccines, including Pfizer‐BioNTech (52.8%), AstraZeneca (20.7%), and Sinopharm (14.2%). About 72% of participants experienced postvaccination side effects. In addition to psychological aspects, menstrual dysfunctions following the COVID‐19 vaccination could be attributed to the inflammatory mediators that the human body produces in response to receiving a vaccine.
Yandong Cheng [44] (2022)	18–50	*N *= 1264	Cohort study	1‐Pfizer	Menstrual change (45%) Menstrual delay (11%) Early menstruation (0/9%) Prolong period (0/3%) Metrorrhagia (0/3%).	Irregular menstrual changes have also been observed in women vaccinated with other types of Covid‐19 vaccines.
Mersal E [45] (2022)	16–40	*N *= 731	Cohort study	1‐Pfizer	Menstrual delay (60/5%) Early menstruation (30/4%) Hypermenorrhea (43/9%) Decreased menstrual flow (43/3%) change in the timing (9/1%) dysmenorrhea (62/4%).	Overall, 50.9% of participants reported a change in menstruation after vaccination, especially those who received 2 doses. There was a positive and significant correlation between the number of doses and the experience of menstrual changes associated with 2 doses.
Nagla El‐Shitany [46] (2021)	> 18	*N* = 1560	Cross‐sectional	1‐Pfizer‐ 2‐BioNTech 3‐AstraZeneca	Menorrhagia (0.42%) Metrorrhagia (0.26%) [Pfizer‐BioNTech] (0.44%) [AstraZeneca] Mean = 0.33%	Pfizer‐BioNTech induced more severe tolls than AstraZeneca AstraZeneca vaccine induces more severe tolls after the second dose.

## 3 Discussion

This study aimed to assess menstrual disorders post‐COVID‐19 condition symptoms in reproductive‐age women and post‐reproductive age. Public reports of the complications and consequences of the COVID‐19 vaccines that alter women's menstrual periods have been published on the Internet and among the public and doctors' offices since the beginning of 2021. Studies have been collected from different geographical regions around the world. A review of studies related to menstrual disorders and COVID‐19 vaccination showed that the prevalence of menstrual disorders among women is 100%. There is heterogeneity between the results of the conducted studies, but all of them have caused disorders in some way, although this relationship was not statistically significant in some cases. Twelve cohort studies and nine cross‐sectional studies have confirmed the association between COVID‐19 vaccination and menstrual disorders. Four studies out of 27 studies have rejected this relationship [3, 4], [17, 18] and 2 studies have reported a mild and short‐term relationship and transient complications [14, 19] It should be noted that 25 studies have been conducted on a combination of different types of vaccines, and only 2 studies have exclusively examined the complications of the Pfizer vaccine. most of the studies reported menstrual disorders such as menorrhagia, oligomenorrhoea, metrorrhagia and dysmenorrhea, hypomenorrhea, polymenorrhagia, and menstrual timing disorder. The tool used to conduct these studies was usually a questionnaire based on the Internet, and since all studies are cross‐sectional, it does not clearly show a causal relationship. A few studies have examined this. Most of them reported menstrual disorders such as menorrhagia, oligomenorrhoea, metrorrhagia and dysmenorrhea, hypomenorrhea, polymenorrhagia, and menstrual timing disorder. The tool used to conduct these studies was usually a questionnaire based on the Internet, and since all studies are cross‐sectional, it does not clearly show a causal relationship. In 13 studies, menorrhagia was reported as the most common complication [14, 17, 19, 20, 21, 22, 23, 24, 25, 26] and any type of Covid‐19 vaccine can affect menstrual regularity. It has also been reported in previous studies that HPV, [27] measles, hepatitis, and diphtheria which are part of viral vaccines, cause menstrual disorders [28, 29]. Menstrual disorders have also been reported during the COVID‐19 vaccination. In one of the cohort studies and one of the cross‐sectional studies [1, 4] that women's Menopausal ages have been investigated, postmenopausal bleeding and remonstration have been shown. Vaccines can activate the immune system through a series of inflammatory factors. Vaccines cause the activation of a series of inflammatory factors such as cytokines and interferons, which prevent infectious agents. These inflammatory reactions cause the cyclic breakdown and repair of the uterine endometrium wall through the natural defense cells inhabiting the endometrium, which itself causes menstrual disorders or irregular uterine bleeding [30, 31].

### 3.1 Strengths and Limitations

One of the strong points of this systematic review was the investigation of the complications caused by corona insemination in reproductive‐age women and after menopause. Among the limitations of the study, firstly, the side effects by vaccine type have not been investigated. Second, most studies have not considered confounding variables and underlying disorders affecting the menstrual cycle.

## 4 Conclusion

The tremendous sum of self‐reporting and the generally tall recurrence from the cohort and cross‐sectional consideration appears that there's a doable causal relationship between COVID‐19 immunization and menstrual changes. But to prove this reason for coloration, more studies are needed. Although menstrual change is very common and can have different causes, in this study, many women experienced menstrual disorders after the COVID‐19 vaccination. Menstrual abnormalities can cause concern in women and also debilitate, a woman's modal of life can be temporary effects.

## Author Contributions

**Farideh Pargar:** investigation, project administration, writing – original draft, data curation. **Zeinab Rabiei:** methodology, formal analysis, resources. **Afsaneh Keramat:** supervision.

## Ethics Statement

The authors have nothing to report.

## Consent

The authors have nothing to report.

## Conflicts of Interest

The authors declare no conflicts of interest.

## Transparency Statement

The lead author Afsaneh Keramat affirms that this manuscript is an honest, accurate, and transparent account of the study being reported; that no important aspects of the study have been omitted; and that any discrepancies from the study as planned (and, if relevant, registered) have been explained.

## Data Availability

The study was a systematic review and the information that formed the findings was extracted from the articles listed in Table 1. The data that support the findings of this study are available on request from the corresponding author. The data are not publicly available due to privacy or ethical restrictions. Keramat F/manuscript guarantor.
